# HIV prevention for South African youth: which interventions work? A systematic review of current evidence

**DOI:** 10.1186/1471-2458-10-102

**Published:** 2010-02-26

**Authors:** Abigail Harrison, Marie-Louise Newell, John Imrie, Graeme Hoddinott

**Affiliations:** 1 Brown University, Population Studies and Training Center, and Department of Medicine, Warren Alpert School of Medicine, Providence, RI USA; 2 Africa Centre for Health and Population Studies, University of KwaZulu Natal, Somkhele, South Africa; 3 Centre for Paediatric Epidemiology, Institute of Child Health, University College London, London, UK; 4 National Centre in HIV Social Research, University of New South Wales, Sydney, Australia

## Abstract

**Background:**

In South Africa, HIV prevalence among youth aged 15-24 is among the world's highest. Given the urgent need to identify effective HIV prevention approaches, this review assesses the evidence base for youth HIV prevention in South Africa.

**Methods:**

Systematic, analytical review of HIV prevention interventions targeting youth in South Africa since 2000. Critical assessment of interventions in 4 domains: 1) study design and outcomes, 2) intervention design (content, curriculum, theory, adaptation process), 3) thematic focus and HIV causal pathways, 4) intervention delivery (duration, intensity, who, how, where).

**Results:**

Eight youth HIV prevention interventions were included; all were similar in HIV prevention content and objectives, but varied in thematic focus, hypothesised causal pathways, theoretical basis, delivery method, intensity and duration. Interventions were school- (5) or group-based (3), involving in- and out-of-school youth. Primary outcomes included HIV incidence (2), reported sexual risk behavior alone (4), or with alcohol use (2). Interventions led to reductions in STI incidence (1), and reported sexual or alcohol risk behaviours (5), although effect size varied. All but one targeted at least one structural factor associated with HIV infection: gender and sexual coercion (3), alcohol/substance use (2), or economic factors (2). Delivery methods and formats varied, and included teachers (5), peer educators (5), and older mentors (1). School-based interventions experienced frequent implementation challenges.

**Conclusions:**

Key recommendations include: address HIV social risk factors, such as gender, poverty and alcohol; target the structural and institutional context; work to change social norms; and engage schools in new ways, including participatory learning.

## Background

With South African youth aged 15-24 experiencing among the highest HIV prevalence in the world [1], the development of effective HIV prevention programmes is a top public health and policy priority [2]. However, in spite of recent calls to increase attention to the high levels of HIV transmission to young women [3], particularly in southern Africa [4], little scientific consensus exists about how best to prevent HIV infection among youth. In countries where HIV prevalence has declined at population level, sexual behaviour change among young people has been cited as an important contributing factor [2]. Yet questions remain regarding how to achieve - and maintain - the individual-level behavioural changes needed to reduce HIV incidence.

Comprehensive sexuality education is considered an important means of addressing adolescent risk behaviours [5,6], although little evidence supports its direct impact on biological measures of prevention success, particularly HIV and other sexually transmitted infections (STIs) [5-7]. In sub-Saharan Africa, experience with youth HIV prevention programmes is limited, with evidence regarding effectiveness still emerging. Recent trials of youth HIV prevention interventions have achieved mixed results. Three large community trials of comprehensive approaches to youth HIV prevention, involving schools and other key institutions and stakeholders, have failed to significantly reduce HIV incidence in young people, and have shown only modest success in increasing protective behaviours [8-10]. However, two group-based interventions in South Africa have shown promise in reducing reported HIV-related risk behaviours, and in one case, associated biological outcomes [11-13]. Both interventions addressed HIV-related structural factors, or the social influences underlying HIV risk [14], namely gender-based violence [11-13] and women's poverty [12,13]. Together with limited results of several smaller, school-based interventions, these outcomes have triggered debate about 'which interventions work' [15].

Prior reviews of youth intervention studies in both developed and developing countries [2,5,7,16-19] suggest an important role for school-based interventions in increasing young people's knowledge of sexuality, reproductive health, and HIV prevention, with a majority leading to reductions in reported risk behaviours [5]. Reviews of school-based interventions specific to sub-Saharan Africa have found greater intervention impact on HIV-related knowledge and attitudes than on reported sexual behaviours [16,17], a finding reinforced by two recent large-scale trials in Tanzania, the Mema kwa Vijana (MkV) Project, and Zimbabwe, the Regai Dzive Shiri (RDS) Project [9,10]. These trials impacted knowledge and attitudes (both), self-efficacy (RDS), some aspects of men's sexual behaviour (MkV), and also self-reported pregnancy (RDS). Lessons learned from these trials include the need to change population norms regarding sexual risk behaviours, and to address a broad range of interpersonal, cultural and structural factors underlying HIV risk [20]. Further, strengthening and broadening existing approaches, like school-based programmes, and adopting new approaches to reduce youth HIV incidence in sub-Saharan Africa are important [16,17,20].

To understand current evidence for youth HIV prevention in sub-Saharan Africa, and to answer the question 'which interventions work, and why', reviews of intervention content and characteristics of successful interventions are needed. While several systematic reviews of HIV prevention interventions have included developing country adolescents [2,5,16-18,21], only three focused on sub-Saharan Africa specifically [16,17,21]. Two reviews included only school-based studies [16,17], while the third reviewed studies with an HIV endpoint [21]. Further, many reviews primarily consider methodological and study design issues [16-19]. Reviews may include few details about interventions, or on secondary and process outcomes that indicate pathways to intervention outcomes. Lastly, since systematic reviews are often based on published studies, recently-completed studies may be excluded, although these may offer relevant lessons learned, helping to identify promising approaches.

The debate about 'which interventions work - and why' has been renewed following recent trial results. In South Africa, the continued severity of the HIV epidemic has lent new urgency to these questions - and their answers [22]. The development of interventions specific to the South African context is an urgent research priority [23], and may support intervention approaches for sub-Saharan Africa more broadly. To inform the development of an evidence-based, state-of-the-art approach to youth HIV prevention in South Africa, we undertook a review of ongoing or recently completed intervention studies, with the aim of systematically assessing characteristics of rigourously designed youth HIV prevention interventions, to better understand how they work, and why.

## Methods

This review addresses study design, intervention design, including content and theoretical basis, thematic focus and HIV causal pathways, and intervention delivery and implementation, among youth aged 12-24 years in South Africa. The review methodology was adapted from other reviews of adolescent HIV prevention studies, to ensure rigorous and systematic assessment of the strength of evidence on intervention effectiveness [2,5,18,19,23].

### Inclusion Criteria

'Rigorously designed' programs were defined as those focused specifically on preventing or reducing HIV-related risk behaviours in young people, or their determinants, or incidence of HIV or other sexually transmitted infections. Selection criteria included: 1) intervention conducted in South Africa, in 2000 or after (since interventions prior to that date focused primarily on increasing knowledge and changing attitudes, and have been included in other reviews); 2) behavioural intervention focused on youth, using a broad definition of 12-24 years of age; 3) experimental design including a control or comparison condition; 4) biological or behavioural outcome, or both; and 5) available information on intervention structure (eg., curriculum, group vs individual format, number of sessions, implementation/delivery personnel), content (eg., major themes and topics, information provided, targeted risk/protective factors), and theoretical framework. To consider the most up-to-date evidence, we included three types of studies: 1) completed interventions with published findings; 2) completed interventions with unpublished evaluations; and 3) promising approaches, which were ongoing interventions that met other criteria, with sufficient data available to assess the intervention's content and likely impact. The result is a systematically conducted review of current youth HIV prevention interventions in South Africa.

The search strategy included computerised searches of MEDLINE, Social Sciences Index, and the NIH search engine CRISP; abstracts from the 2004, 2006 and 2008 International AIDS conferences; examination of already-published systematic reviews [16,17], and expert consultation, creating a 'snowball' effect when other appropriate interventions were identified. Search terms included "schools, HIV prevention, South Africa" and "youth, HIV/AIDS, South Africa." We distinguished between 'programmes' and 'interventions', and excluded national programmes, such as LoveLife or Soul City [24,25], as well as programmes designed to support the national LifeSkills programme and their evaluations [26-28], since these tended to be larger, with a geographically broader target population, and often had multiple components that rendered the evaluation of impact in a specific population more difficult. Instead, the review included interventions that were curriculum-based, or followed a similar structured protocol, aimed at effecting behavioural changes leading to a decrease in HIV incidence or related risk behaviours among South African youth. Some print media or health communication evaluations not explicitly designed to measure behaviour change were also excluded [29].

### Selection of Studies

Nine intervention studies were initially identified [11,13,30-36]. After further review, one of the nine - although a promising model based on a community participatory approach - was excluded as it was a family intervention targeting parents, caregivers and pre-adolescents, and did not measure impact in youth aged 12-24 [30]. One of the eight selected was not formally a 'youth' intervention, but included an evaluation of the program's impact on young women and was thus included, although results need to be interpreted cautiously as the study was not designed with sufficient power to measure outcomes in this sub-group [13]. Following identification of the studies, informal interviews were conducted with at least one researcher from each selected intervention, and requests made for copies of project materials. The process was further informed by an expert scientific workshop convened to discuss state-of-the-art issues related to schools and youth HIV prevention research.

### Analytical Process

Information from these sources, as well as published reports, was used to prepare a description of each intervention, highlighting the substantive focus and hypothesised pathways to achieving the study's primary outcome(s). The main analytical categories were specified *a priori*, based on characteristics of interventions demonstrated to be successful in diverse settings [5,16,17]. These included: 1) process of intervention development, including formative research; 2) cultural/linguistic adaptation; 3) use of social/behavioural theory; 4) how and where the intervention was delivered (eg. classroom, community, after school, extra periods); 5) who delivered the intervention (eg., peer educators, teachers, trained facilitators); 6) selection and reinforcement of key messages; 7) involvement of participants and/or broader community; 8) focus on social context and risk environments, as well as individual risk behaviours; and 9) focus on HIV causal pathways of relevance to South African setting.

## Results

### Study Design and Outcomes

Eight interventions were included in the review. Stepping Stones, which reduced incidence of HSV-2, was one of two studies to measure biological outcomes, and the only one to report a significant impact [11]. Five of eight interventions demonstrated a significant improvement in reported HIV-related risk behaviours [11,13,32,33,35], including condom use [33], HIV testing [13,35], heavy drinking [11] or alcohol use during sexual activity [32], and intimate partner violence [11,13]. Two other interventions resulted in maintenance of reported risk behaviours in the intervention group, with increased reports of sexual or alcohol risk behaviour in the comparison group [34,36], while one intervention showed no effect at all [31] (Table 1). Although all studies employed a control or comparison group, experimental designs varied widely, with some having non-randomised selection, multiple versus single assessments, sample sizes ranging from several hundred to several thousand participants, differences in length of follow-up period, active versus passive controls and cluster versus individually-randomised designs (Table 1). These differences contributed to a range of effect sizes, making comparison of intervention outcomes and impact difficult (Table 2). Further, assessment methods differed: surveys were conducted using electronic data capture via PDA (personal digital assistant) [31], self-administered classroom surveys using pen and paper [32,33,35,36], or individual assessment by interviewers [11,13,34]. All interventions conducted 'process' evaluations - generally qualitative - to monitor intervention implementation (Table 1), and several studies had additional published reports on details of study design [37-40] or qualitative assessments [41,42].

**Table 1:** Study Design for Eight Youth HIV Prevention Interventions in Systematic Review

Project Description	Objective	Target Population/Age	Experimental Design & Sample Size	Control or Comparison Condition	Duration of Follow-up
**HAPS **[32] **[HIV/AIDS Prevention Study] **KwaZulu/Natal	Reduce sexual and alcohol risk-taking behaviors	Secondary School students, Grade 9; Ages: 14-16 years	Random assignment at school level; 3 intervention, 2 comparison schools N = 325 I; N = 336 C; pre-post survey	Standard life skills/life orientation curriculum	Follow up survey: 2 months post-intervention

**HealthWise **[34] CapeTown	Reduce STI/HIV transmission, drug/alcohol abuse and increase positive use of leisure time	Secondary school students, Grades 8-9 Ages 12-14 in urban township setting;	Pre-and post-intervention surveys; in 3 8^th ^grade cohorts; 4 intervention, 5 control schools randomly assigned; N = 901 I; N = 1275 C	Standard life skills/life orientation curriculum	5 waves of data collection for each cohort over 1.5 years

**Mpondombili Project **[33] KwaZulu/Natal	Promote safer sex behaviors, with emphasis on *dual protection*, sexual risk-reduction, and promotion of positive gender role norms	Secondary School students: Grades 8-10 in rural secondary schools	Baseline and follow up surveys in 2 intervention and 2 comparison schools, not randomized; N = 442 I; N = 541C	Standard life skills/life orientation curriculum; comparison schools received shortened version of curriculum (delayed)	Follow up survey: 5 months post-intervention

**Adolescent Livelihoods**[35] KwaZulu/Natal	Reduce HIV risks and social vulnerabilities, increase access to 'safe spaces' and life skills	Urban township; Out of school youth aged 16-24; in-school youth aged 14-20	Quasi-experimental; group assignment		2 years

**SATZ **[31,37,51,52] CapeTown and Northern Province	To develop, implement and evaluate a school-based health education program aimed at promotion of correct, consistent condom use and delay in sexual debut	School students in urban township and rural area; ages 12-14 in grade 8	1 pre- and 2 post-test assessments within quasi-experimental design; 13 intervention and 13 control schools; not randomized. N = 3625	Comparison schools received delayed intervention	1 year

**Stepping Stones**[11,38] Eastern Cape	Promote sexual and reproductive health via HIV prevention and reduction in sexual coercion and intimate partner violence	Semi-urban township; older adolescents and young adults aged 18-24; in- and out-of-school youth	Cluster RCT; matched control group; 35 I clusters; 35 C clusters. Sample size: 2770 N = 1140 I; N = 1081C	Single session on HIV, condoms, safe sexual behaviors	2 years post-intervention with 2 assessments, at 12 months and 24 months

**Tshwane Peer Education and Support Programme**[36] Tshwane (Pretoria), Gauteng	Promote accurate information about HIV/AIDS, address peer norms, establish psychosocial support	High school students ages 13-20, in semi-rural secondary schools	13 intervention and 4 control schools; not randomized. Pre-post survey of one selected class in each school. N = 1572 I; N = 596 C	Ongoing Life Orientation or other HIV prevention activities	18 months

**IMAGE - Intervention with Microfinance for AIDS and Gender Equity**[12,13]	To evaluate effects of combined microfinance and training intervention on HIV risk behavior	Of 3 evaluation cohorts, one cohort of 14-35 year old women	Cluster RCT; 8 pair-matched villages. N = 130(I); N = 132 (C)	Villages randomised to control received standard of care; available sexual health info; no microfinance	2 years

**Table 2:** Study Results for Eight Youth HIV Prevention Interventions included in Systematic Review^1^

Project Name and Location	Impact of Intervention on: Knowledge, Attitudes, Perceptions, Social Norms	Behavioral skills and intentions for risk reduction (communication, negotiation, self-efficacy)	Sexual and other HIV risk behaviours	Clinical and biological outcomes and/or Structural and Community Effects
**HAPS **[32] **[HIV/AIDS Prevention Study] **KwaZulu/Natal NB: Study results presented as scores from scaled measures; analyzed using ANOVA for each group, and showing net intervention effect for difference between intervention (I) and comparison (C) groups	Positive attitudes to condom use 0.17I vs 0.08C, interv effect: 0.09 Positive attitudes toward alcohol -0.01I vs 0.16C, interv effect: -0.17 Negative attitudes toward alcohol -0.15I vs -0.08C, interv effect: -0.07	Self efficacy for sex refusal 0.08I vs- 0.16C, intervention effect: 0.24 Self efficacy for condom use -0.08I vs -0.06C, intervention effect: -0.02 Self-efficacy for alcohol refusal 0.17I vs 0.44C, intervention effect: -0.27 Condom use intentions: 0.18I vs -0.01C, intervention effect: 0.19 Intentions to have sex - next 3 months: 0.00I vs 0.06C, intervention effect: -0.06	**Alcohol Behaviors**: Frequency of alcohol use: last 14 days 0.23I vs 0.28C, intervention effect: -0.05 Number of drinks last time drinking 0.36I vs -0.08C, intervention effect: 0.44 Self or partner drinking at last sex -0.9I vs 4.5C, intervention effect: -4.4 **HIV-related behavior:** Condom use at last sex 4.2I vs 2.2C, intervention effect: 2.0	Not measured

**HealthWise **[34] CapeTown NB: Assessment of intervention conducted in 5 waves of data collection; unless otherwise specified, results are presented as comparison between intervention (I) and comparison (C) groups at last followup (wave 5)	Non-users of alcohol, cigarettes, and marijuana at wave 1* Lifetime Use Alcohol: 62%I vs 60%C, OR = 0.9 [0.7-1.2] Cigarettes: 39%I vs 45%C, OR = 1.2 [0.9-1.6]^3^ Marijuana: 45%I vs 45%C, OR = 1.0 [0.8-1.2]** Use in past month: Alcohol: 22%I vs 29%C, OR = 1.4 [0.99-2.0]^3^ Cigarettes: 28%I vs 35%C, OR = 1.4 [1.04-1.8]^3^ Marijuana: 18%I vs 15%C, OR = 0.8 [0.6-1.1]** Heavy use: Alcohol: 8%I vs 12%C, OR = 1.7 [1.04-2.6]^3^ Cigarettes: 13%I vs 17%C, OR = 1.4 [0.9-1.9]^3^	**Sexual risk:** Perception of condom availability (condom access: can get condoms): 95%I vs 92%C, OR = 1.6 [1.03-2.4] Alcohol, Smoking and Marijuana* Alcohol use in: Past month: 32%I vs 39%C, OR = 1.4 [1.1-1.8]^3^ Heavy use: 13%I vs19%C, OR = 1.6 [1.2-2.2]^3^ **Cigarette use in**: Past month: 41% vs 48%, OR = 1.4 [1.1-1.7]^3^ Heavy use: 22%I vs 28%, OR = 1.4 [1.1-1.8]^2.3^ Marijuana use in past month: 22%I vs 18%C, OR = 0.8 [0.6-1.1]** **in males, significantly higher reported use in intervention group	**Sexual Behavior** Sexually active (lifetime) among those not sexually experience at baseline: 22%I vs 21%C, OR = 1.0 [0.8-1.3] **Frequency of**: Sexual activity (past month)***: -6 I [-19 -+6] vs -2C [-12 - +8] Condom use (always)**: 0 I [-12 -+12] vs +2C [-9 -+12] *Since knowledge and attitudes of alcohol and other substances were not reported, these columns report data on use behaviors in those experienced (column 2) and not experienced (column 1) at baseline. ***reported as 'change in prevalence' between waves 4 and 5 of assessment	Not measured

**Mpondombili Project **[33] KwaZulu/Natal NB: *denotes significance at p < 0.05 level, based on analysis of data using regression methods for comparison of I and C groups at follow up.	General HIV Knowledge^3^: 67.3%I vs 54.7C, beta coefficient: .26 [-4.30 - +4.35]* Know where to access HIV test^2^: 76.6%I vs 62.6%C, beta: 0.57 [0.08-1.05] Peer norms for abstinence (mean score): 3.38I vs 3.67C, beta: 0.28 [-0.04-0.59]* Peer norms for girls' condom use (mean): 2.42I vs 2.24C, beta: -0.07 [-0.27-0.12] Egalitarian beliefs about sex refusal: 6.27I vs 6.07C, beta: 0.38 [-0.20-0.97]	Self-efficacy for sex refusal^3^: 84.8%I vs 77.8%C, beta: 0.49 [0.25-1.24]* Self-efficacy for condom use^3^: 93.8%I vs 87.0%C, beta: 0.61 [0.06-1.16]* Communication with partner about condoms^3^: 74.7%I vs 57.5%C, beta: 2.18 [1.21-3.91]* Perceived risk for HIV: 90.8%I vs 86.5%C, beta: 0.09 [-0.40-0.58] Perceived risk for pregnancy: 57.8%I vs 49.2%C, beta: 0.19 [-0.16-0.54]	Ever condom use^3^: 67.3% I vs 41.9%C, OR = 2.85 [1.62-5.04]* Condom use at last sexual intercourse^2,3^: 54.3%I vs 27.9%C, OR = 3.21 [1.76-5.85]* Ever had sex^2^: 28.5%I vs 22.2%C, OR = 1.39 [1.02-1.91]*	Not measured

**Adolescent Livelihoods **[35] KwaZulu/Natal NB: Preliminary findings. No measures of significance provided.	**Indicators of financial well-being** Has savings: M: 34%I vs 34%C; F:27%I vs 8%C Used financial services from a bank: M: 49%I vs 32%C; F:17%I vs 3%C Discussed financial decisionmaking M: 56%I vs 23%C; F: 75% I vs 21%C	**Partner communication in last year** Self-esteem: M: 51%I vs 23%C; F: 70%I vs 26%C Delay/avoid sex in last 12 months F: 76%I vs 66%C Sexuality M: 49%I vs 19%C; F: 67%I vs 26%C Contraception M: 49%I vs 19%C; F: 60%I vs 29%C Violence/Sexual Abuse M: 51%I vs 21%C; F: 62%I vs 24%C Condom use M: 56%I vs21%C; F: 75%I vs 29%C	**Among women:** Reported HIV testing: 34% baseline vs 57% post-I Discussed avoiding STDs with partner: 91%I vs 89%C Discussed avoiding HIV with partner: 87%I vs 77%C	Not measured

**SATZ **[31,37] CapeTown and Northern Province NB: With regard to outcomes, preliminary findings only are available. Process evaluations have been published[51,52].	Knowledge, Attitudes, Social Norms, Self-efficacy were measured. Knowledge increased in intervention schools	**No intervention effects **observed. No effects on sub-groups or for secondary outcomes.	**Sexual Risk Behavior** Ever had sex: 25.6%I vs 24.6%C, OR = 1.0 [0.85-1.18] Transition to sexual activity: 19.0%I vs 16.7%C, OR = 1.05 [0.85-1.29] Condon use at last sex: 50.2%I vs 44.9%C, OR = 1.13 [0.79-1.62]	Not measured

**Stepping Stones **[11,38] Eastern Cape NB: As knowledge, attitudes and related measures were not reported in main outcome paper, these categories are not included here. Results are from 24 month follow-up.	Correct condom use at last sex M: 73.2%I vs 75.1%C, OR = 0.88 [0.64-1.21], p = 0.43 F: 57.5%I vs 59.6%C, OR = 0.90 [0.70-1.17], p = 0.45 Problem drinking: M: 26.6%I vs 25.7%C, OR = 1.1 [0.81-1.49], p = 0.56 F: 3.4%I vs 2.2%C, OR = 1.4 [0.61-3.17], p = 0.43 Ever misused drugs M: 6.5%I vs 12.0%C, OR = 0.50 [0.23-1.11], p = 0.88 F: 2.3%I vs 1.9%C, OR = 1.2 [0.51-2.83], p = 0.68 Depression M: 2.8%I vs 5.0%C, OR = 0.52 [0.24-1.13], p = 0.56 F: 12.8%I vs 16.1%C, OR = 0.76 [0.51-1.15], p = 0.20	Number of partners (past year): M (mean): 2.15I vs 2.39 C, effect: -0.0045 [-0.023-0.0003], p = 0.12 F: (mean): 1.19I vs 1.19 C, effect: 0.0001 [-0.0012-0.0025], p = 0.73 Transactional sex w/casual partner M: 1.8%I vs 1.9%C, OR = 1.02 [0.39-2.65], p = 0.031 F: 2.0%I vs 2.2%C, OR = 0.94 [0.41-2.18], p = 0.89 >1 incident intimate partner violence M: 6.2%I vs 96%C, OR = 0.62 [0.38-1.01], p = 0.054 F: 14.7%I vs 13.5%C, OR = 1.12 [0.77-1.68], p = 0.51 Any casual partner M: 53.1%I vs 56.9%C, OR = 0.85 [0.62-1.13], p = 0.29 F: 18.3%I vs 16.3%C, OR = 1.17 [0.85-1.63], p = 0.34		**Biological** HIV incidence: F: 5.65I vs 6.95C M: 1.4I vs 1.29C, p = 0.78 HSV-2: F: 5.35I vs 7.71C M: 1.46I vs 2.04C, p = 0.36 Pregnancy F: 14.4I vs 11.6C, OR = 1.45 [0.92-2.28], p = 0.11 M: 11.3I vs 12.9C, OR = 0.88 [0.60-1.31], p = 0.53

**Tshwane Peer Education and Support Programme **[36] Tshwane (Pretoria), Gauteng NB: In published results, measures of significance were based on 'within group' comparisons (eg., pre- and post-test). For consistency, we present 'between group' comparisons of the post-test measures in I (n = 1572) and C (n = 532) groups.	Perception of peer sexual activity (most friends having sex) 20.7%I vs 25.4%C Friends practice safe sex 51.0%I vs 54.2%C Most friends drink alcohol 25.0%I vs 27.2%C	Findings reported as difference in scale scores: Psychological well-being: 52.44I (SD6.8) vs 51.51C(SD6.8) Personal control: 7.48I(SD2.1) vs 8.10C(SD2.1) School Climate: 57.49I(SD11.4) vs 58.31C(11.7)	Sexual experience: 41.6%I vs 46.2%C Sex during past 3 months: 36.9%I vs 30.8%C Multiple partners in past 3 months 15.6%I vs 17.3%C Condom use every time in last 3 months: 59.3%I vs 54.7%C Sex without consent 19.7%I vs 20.8%C Current Alcohol Use 21.9%I vs 22.7%C Excessive alcohol use 13.1%I vs 18.0%C Illicit drug use 6.5%I vs 7.2%C	Not measured

**IMAGE - Intervention with Microfinance fir AIDS and Gender Equity **[12,13]	Knowing HIV+ person can look healthy: 91% I vs 87%C, RR = 1.09 [0.73-1.62] Comfort discussing sexuality in the home: 84%I vs 68%C, RR = 1.22 [0.53-2.80]	Communication in h'hold about sex (past year): 74%I vs 50%C, RR = 1.46 [1.01-2.12]	>1 sexual partner in past year: 4%I vs 3%C, RR = 0.95 [0.40-2.27] Unprotected last sex, non-spousal partner: 55%I vs 78%C, RR = 0.76 [0.60-0.96] ?HIV test: 29%I vs 18%C, RR = 1.64 [1.06-2.56]	No biological outcome measures for analysis of women under age 35

^1^All data are presented for combined male and female results, except where published results included only gender-disaggregated results (eg., Stepping Stones and Adolescent Livelihoods projects). ^2 ^Indicates significant findings for sub-sample of males in Intervention vs Control at followup (not shown). ^3 ^Indicates significant findings for sub-sample of females in Intervention vs Control at followup (not shown).

### Intervention Design

Five interventions were school-based [31-34,36], and employed a standard classroom approach to intervention delivery, while the remaining three were group-based [11,13,35] and delivered in community venues or schools after-hours (Additional file 1: Table S3). All eight interventions included broadly similar information about HIV transmission and prevention, although relative emphasis on condoms and abstinence/delay of sexual activity, and related topics such as sexual negotiation, gender issues and sexual coercion, self esteem and interpersonal communication, and drugs and alcohol varied. Several interventions addressed pregnancy and contraception, while a few emphasized partner reduction and safe partnerships, and HIV testing [11,13,31,33]. Two interventions strongly emphasized broader life skills, including numeracy [35], or training in economic support [13,35]. With the exception of the IMAGE and HAPS projects [13,32], all interventions were single-component (Additional file 1, Table S3). Some interventions incorporated multi-level approaches, as in the two curricula in HAPS addressing sexual risk reduction and alcohol [32]. Two other projects [34,35] addressed multiple, discrete topics within one intervention, such as HIV prevention and financial skills [35], HIV and pregnancy prevention, or 'dual protection' [33], sexual and alcohol risk reduction coupled with positive leisure time use [34], or school-level factors [36]. All interventions, both school- and group-based, used written curricula, except for the TPE Project, in which facilitators and peer educators developed role plays and presentations.

### Intervention Development and Cultural Adaptation

Considerable variation existed in the cultural adaptation of interventions to South Africa. Five curricula were developed in the US or UK, while Stepping Stones was developed in Uganda. These were adapted via qualitative research [31,32] or participatory action research [11,13,33,34] with the target audience. One of the five adapted specific modules from several effective interventions in the US and South Africa [33], rather than entire curricula [11,31,32,34]. Two interventions were developed locally [35,36], with one not using a formal curriculum [36].

### Theoretical Framework

Most studies were based on social cognitive models of behavioural change [31-34,36], although possible limitations of these individual risk-reduction models for non-Western contexts were acknowledged [31,33] (Additional file 1, Table S3). To address this, some studies expanded existing theoretical frameworks to include socio-cultural factors [31], or used individual and group-level theories together [33]. Three studies used an empowerment model based on the theories of Paolo Freire [11,13,33].

### Thematic Focus and HIV Causal Pathways

Each intervention had a unique thematic focus within overall HIV prevention goals (Table 1), reflecting the hypothesised causal pathways to HIV infection. Generally, the causal pathways represented probable influences on HIV infection and associated risk factors. Stepping Stones and IMAGE, for example, addressed gender-based violence as an important influence on HIV risk, with reduction of intimate partner violence a key intervention message [11]. HAPS and HealthWise both strongly emphasized alcohol use as a risk factor for HIV infection [32,34]. Along with Stepping Stones and IMAGE, Mpondombili focused on gender equity, adopting an empowerment approach to challenge negative gender values [33]. Adolescent Livelihoods and IMAGE focused on the economic context of HIV risk, specifically the attainment of vocational or financial life skills aimed at individual empowerment [13,35]. In addition to peer social norms related to HIV prevention, the TPE Project [36] addressed school-level factors that could support students' HIV prevention goals, while HealthWise focused on positive leisure time use [34]. While these are not, with the exception of IMAGE, truly 'structural interventions', they are focused on key structural factors that affect HIV risk at the individual level, as well as their social context.

### Intervention Delivery

Five interventions used standard, classroom-based methods [31-34,36]. Some studies used schools in innovative ways, including Healthwise's after-school activities [34], or as a meeting place for in-school and out-of-school youth in Adolescent Livelihoods [35], and for extra-curricular sessions in Stepping Stones [11]. The main differences, however, between the school-based and non-school-based approaches were the individual versus group approaches. Importantly, the use of group-based intervention delivery appears to have fostered positive shifts in group norms regarding HIV risk behaviours [11,13,35].

### Who Delivered the Intervention?

In 4 of the 5 school-based interventions [31-34], teachers had a main role in delivering the intervention. The SATZ and Mpondombili projects trained peer educators to co-facilitate the intervention with teachers in classrooms [31,33]. The TPE Project relied on peer educators with guidance from post-graduate student advisors, and the support, but not active involvement, of teachers [36]. Teachers generally were responsible for overseeing peer educators as well as the curriculum, with assistance from an intervention team [31-33].

Peer educators of the same age or slightly older participated in all but one school-based intervention [31-33,36]. Several interventions had older peer facilitators, who functioned as mentors and were responsible for leading structured group discussions [11,35]. Usually, peer educators were selected via a competitive process [9,33], or via nomination by student peers [32,36]. In some projects, such as TPE, teachers made the final selection [36]. Training generally consisted of strengthening the peer educators' knowledge base, and developing facilitation, interpersonal and public speaking skills. Length of training varied, however, from a 1-2 day workshop in the Tshwane and HAPS projects, to six months in Mpondombili. In HAPS, peer leaders led discussions following audiotaped vignettes about teenagers' dilemmas regarding alcohol use and sexual activity [32]. In Mpondombili, as well as HAPS, SATZ and HealthWise, teachers also received extensive training, ranging from 2-3 days to several multi-day workshops (Additional file 1, Table S3). Overall, peer educators were popular with students, but required high levels of training and support for their expected roles.

### Intervention Duration, Intensity and Reinforcement of Key Messages

The eight interventions varied greatly in number of sessions, programme duration, and frequency of sessions (Additional file 1, Table S3), ranging from 8 weeks to six months, with two interventions providing booster sessions [31,34]. The number of intervention modules ranged from 10-16, although there was a wider range in the number or sessions, as some modules took 2-3 sessions to complete [32,34]. In-school and out-of-school interventions were of similar duration.

### Summary of Review Findings

In summary, the review finds that, although within these trials the effects of most interventions on reported sexual risk behaviour or biological outcomes were limited, common elements related to their impact on secondary outcomes can be discerned, as well as aspects of intervention delivery. These include: 1) a focus on at least one social/structural risk factor, as in the emphasis on gender, poverty and alcohol in these eight interventions, 2) using group-based delivery to change social norms, 3) within schools, demonstrating the need to use additional personnel, perhaps from outside the school setting, to deliver interventions, thus relieving a burden on teachers, and 4) directing intervention efforts at the school, as well as individual, level. In addition, several studies demonstrate the potential of structural interventions to bring about changes in HIV-related risk behaviours.

## Discussion

How do the findings from this review answer the question 'what works' to prevent HIV infection in young people in southern Africa? Importantly, the review identifies key elements associated with intervention impact, and highlights promising approaches in youth HIV prevention for South Africa and similar settings. These eight interventions, most of them rigorously designed with regard to intervention and evaluation, indicate progress toward improved HIV prevention, based on behavioural proxy measures for HIV infection. However, given the range of interventions that have been tested, differences in the relative strengths and weaknesses of evaluation designs, and the limited effectiveness of many interventions, a definitive assessment of 'what works' is not possible. Yet important lessons are learned, leading to specific recommendations for future research.

What are the lessons learned from the eight studies included in this review? **First**, moving beyond individual-level measures of knowledge and psychosocial factors to address social and structural factors underlying HIV risk is the main success of these interventions. An important feature - and their area of greatest impact - was the focus on HIV causal pathways relevant to southern Africa, namely gender, sexual coercion, alcohol use and economic risk. Results of the two other major youth intervention trials in sub-Saharan Africa, MkV and RDS, support the importance of addressing social and institutional, as well as individual level, factors to change population-level norms about HIV risk behaviours [9,10,21]. However, addressing social factors is not the same as implementing a 'structural intervention', as in the IMAGE project where women's access to economic resources apparently increased personal empowerment, leading to reduced sexual risk behaviour [42], a finding observed in the larger trial as well as the sub-group of younger women. Similarly, Stepping Stones led to changes in gender beliefs and values, thus impacting the structural context of risk [11]. An important **second **lesson, then, is the need for interventions to adopt structural approaches that can alter the context of young people's HIV risk. Although structural interventions are often critiqued as 'social development' rather than focused health interventions [14], in fact these studies offer several important examples of how targeted structural approaches can change individual behaviour. The HealthWise intervention offered young people alternatives for leisure time use, while in the Adolescent Livelihoods project young people learned life skills, such as numeracy, that may enhance vocational and educational success. Promising economic interventions are also being tested in other sub-Saharan African settings [43]. **Third**, changing social norms related to HIV risk and protective behaviours is important. Stepping Stones' success was clearly associated with altering beliefs about gender and HIV risk, particularly among men, and with offering viable alternative normative behaviours [11,44]. One way that group intervention approaches, such as IMAGE and Stepping Stones, generate positive social norms is through engaging participants in collective critical thinking, thereby fostering self-esteem and individual empowerment [45]. Community mobilisation, employed effectively among women in the IMAGE project and in other promising interventions [13,14,45], is also an important component. Importantly, most school-based interventions do not use a group approach, but are delivered didactically by teachers in classrooms, relying on the ability of students to act individually on information received. A simple way to address this would be for school-based interventions to include more group-based, rather than didactic, learning. A **fourth **lesson learned is thus the need to engage schools differently in HIV prevention, including use of personnel other than teachers to deliver interventions. While peer education is popular among students, this review supports other findings that offer little evidence for its ability to increase intervention impact [46,47]. One approach with certain advantages over teachers, who often resist teaching sexuality education, and same-age peers, who may sometimes have difficulty commanding the necessary authority to run a classroom [41,47], would be to use older youth as 'mentors'. School mentors could work in partnership with teachers who request to teach sexuality education and HIV prevention, but would relieve the reliance on teachers who do not want this responsibility.

Of the five school-based interventions reviewed, four experienced serious implementation problems, leading to calls for interventions to target the school level as well as individual students [31,32,34]. Interventions in South African schools experience challenges to fidelity, due to scheduling disruptions, high levels of student and teacher absenteeism, frequent violence and other school-level issues [48]. These findings thus suggest the need to change how school interventions are implemented, with enormous potential to improve on current models. Yet in spite of the difficulties, schools have some important advantages for intervention delivery, including the ability to reach a large number of youth [49,50], suggesting that working to improve school-based intervention models is an important priority. Methods from the education sector could inform HIV prevention interventions, including the use of school mentors, teacher training in participatory learning approaches, and efforts at school-level change [48]. At the same time, recognition of the considerable variation in youth needs is important, and interventions in community or other venues designed to reach out-of-school youth, or older adolescents, are also research priorities.

## Conclusions

Based on the findings from this review - and from the MkV and RDS trials - specific recommendations for a 'third generation' of youth HIV prevention interventions can be made. These include 1) conducting trials of youth-focused structural interventions, with the aim of altering the structural context of HIV risk; 2) developing new approaches for schools, including interventions that target school-level factors and engage schools as active partners, including mobilising the broader 'school community' of students, teachers, parents and community members; and 3) ensuring that future trials have better measurement and more rigorous designs, including HIV incidence - or another comparable biological measure, such as HSV-2 - as the primary outcome, as well as longer-term follow-up. These recommendations can help to address remaining gaps in knowledge about youth HIV prevention, in spite of important lessons learned from the 'second generation' interventions reviewed here. These include a more definitive understanding of the merits of multi- versus single-component interventions, or of broad-based versus more narrowly targeted approaches, and developmental considerations, like age, gender, sexual activity, and schooling status. These recommendations can help to improve youth HIV prevention research, where - in spite of great progress - no interventions have yet demonstrated a reduction in HIV incidence. This remains the most important - and essential - marker of intervention success.

## Competing interests

The authors declare that they have no competing interests.

## Authors' contributions

AH and MLN designed the review, and contributed to all phases of the research and writing. In addition, AH collected and analyzed the data for each study included in the review. JI contributed to analysis and interpretation of results, as well as preparation of the manuscript. GH contributed to data analysis and preparation of the manuscript. All authors reviewed and approved the final version of this manuscript.

## Pre-publication history

The pre-publication history for this paper can be accessed here:

http://www.biomedcentral.com/1471-2458/10/102/prepub

## Supplementary Material

Additional file 1 **Table S3**. Intervention Design, Content and Characteristics for Eight Youth HIV Prevention Interventions included in Systematic Review Click here for file
