# Supplementary material for: HIV prevention for South African youth: which interventions work? A systematic review of current evidence
Source: BMC Public Health. 2010 Feb 26;10:102. doi: 10.1186/1471-2458-10-102 (PMC2843660; doi:10.1186/1471-2458-10-102)
Supplement: Additional file 1 — Table S3. Intervention Design, Content and Characteristics for Eight Youth HIV Prevention Interventions included in Systematic Review [file 1471-2458-10-102-S1.DOC]

**Intervention Design, Content and Characteristics for Eight Youth HIV Prevention Interventions included in Systematic Review**

| **Project Name** | **Curriculum and/or Intervention Details** | **Intervention Development and/or Adaptation** | **Theoretical Framework** | **Duration** | **Intensity: Number & Frequency of Sessions** | **Booster Sessions** | **How Delivered: Teaching Method** | **Where Delivered** | **Who Delivered: Intervention Personnel** | **Process Evaluation** |
| --- | --- | --- | --- | --- | --- | --- | --- | --- | --- | --- |
| **HAPS [HIV/AIDS Prevention Study]^32^** KwaZulu/Natal | Use of *Amazing Alternatives* and *Project Northland* curricula and media-campaign with drama | Adaptation of two US-based curricula, *Amazing Alternatives*, and Project Northland | Theories of health behavior change: social learning theory, social inoculation, cognitive learning theory | 2 months | 10 units, 30 minutes each | N | Participatory learning with interactive techniques, including vignettes, drama and media | Classrooms; Grade 9 Life Orientation period | Teachers and Peer Leaders | Staff visits, observation, formal monitoring system in place |
| **HealthWise^34^** | US-based curricula, *Life Skills Training* and *TimeWise*, focused on risk reduction & positive leisure time activities | Participatory adaptation process involving target population; qualitative research and feedback | Human Development Theory; Multidirectional Influences (MDI) | One school year (Grade 8) plus booster sessions in Grade 9 | 12 lessons in Grade 8, delivered over 2-3 class periods | Y: 6 booster lessons in Grade 9 | Teacher-led participatory learning with learner workbooks; | Classrooms | Teachers; Youth Development Specialists for comm’ty outreach | Ongoing support for teachers and workshops throughout year ^40^ |
| **Mpondombili Project** ^33^ | 15 session curriculum focused on Dual Protection and altering gender role norms | Compiled/adapted from diverse curricula in USA and SA; input to 18 month process from teachers, nurses and students | Empowerment Theory: individual (Zimmerman) and group levels (Freire) | 4 months | Weekly one hour sessions over 16 weeks | N | Participatory learning via role playing, group exercises, modeling of HIV preventive behaviors | Classroom, Grades 9-10 Guidance or Life Orientation periods | Peer educators, Teachers, Nurses | Member of Work Group was present at each lesson; in-depth interviews with key personnel: peer educators, teachers, nurses^41^ |
| **Population Council, Adolescent Livelihoods^35^** | 6 module curriculum specially designed for context, age & socio-cultural group | Locally developed curriculum for knowledge and skills on sexual behavior and financial literacy | Not explicit; livelihoods support framework | 6 months | Weekly sessions over x month implementation | N | Group-based learning with facilitation by young adult program mentors | Out-of-school,; community venues, such as community halls | Older peer facilitators | Focus groups and in-depth interviews |
| **SATZ ^31^** | Sexual Health Education curriculum | Designed using Intervention Mapping method, with curriculum advisor, teachers, research team | Intervention Mapping protocol to develop theory and evidence-based health promotion; social learning theory, with cultural adaptation | 2-3 months | 16 lessons over 25-55 periods (location dependent) | N | Teacher led sessions, along with learner workbooks | Life Orientation classes | Teacher-implemented curriculum | Process Evaluation: Staff observed classrooms, maintained informal contact with teachers; qualitative data collection |
| **Stepping Stones ^11^** | Stepping Stones curriculum for group-based learning; gender transformative | Adaptation of Stepping Stones curriculum developed in Uganda | Community Development and Process of Change (Freire) | 6-8 weeks | Thirteen 3 hour group sessions; approximately 50 hours total | N | Participatory group-based learning, led by trained peer facilitators; role play, drama, critical reflection | On school premises, after hours. | Trained facilitators, slightly older peers | Researchers monitor implementation; qualitative research |
| **Tshwane Peer Education and Support Programme ^36^** | Emphasis on role of peer educator to raise awareness, mobilize youth & facilitate change in group norms | Training and feedback for peer educators; no curriculum | Systems Theory; emphasis on empowering peer educators | One school year; embedded in schools | Peer educators responsible for determining program at school level | N | Peer Education in classrooms: included HIV awareness via plays, speakers, art, AIDS Days; classroom lectures | Classrooms; -intermittent sessions plus peer education offices | Peer educators, with support from post-grad group facilitators & teachers | Focus group discussions with peer educators and teachers |
| **IMAGE – Intervention with Microfinance fir AIDS and Gender Equity^12,13^** | Combined intervention of group-based microfinance with gender & HIV training curriculum, Sisters for Life | Adapted group gender & HIV curriculum, plus micro-lending to women’s groups for small business devm’t | Participatory Learning and Action | 12-15 months | Every 2 weeks | N | 10 one hour sessions of Sisters for Life for women in microfinance intervention, followed by community mobilisation | Community venues | Trained peer facilitators, all women from community | In-depth and key informant interviews with women participants, FGDs with loan group members; observations & diaries^42^ |

**Table 3: Additional Material**
